# Asylum War Hospitals

**Published:** 1920-09-18

**Authors:** 


### THE PUBLIC WELL-BEING.

### Asylum War Hospitals.

### SERIOUS ALLEGATIONS REFUTED.

The White Paper issued by the Government on the administration of asylum war hospitals is particularly interesting in view of the allegations made previously in the Press that ex-soldiers, whose mental condition was a direct consequence of war service, had been confined in unlicensed and uninspected lunatic asylums and subjected to harsh treatment. Sir Marriott Cooke and Dr. Herbert Bond, Commissioners of the Board of Control, refute these allegations in their report.

The Board are confident that the supervision in all these war hospitals and institutions has been so strict, and that the patients have had such opportunities of daily access to the medical officers and others of the higher officials that systematic ill-treatment has been and is impossible without its speedily becoming known and being severely dealt with.

"At the same time it has to be remembered that there are some insane patients who quite unknowingly misinterpret events that are passing around them, such as, for example, attributing the struggles of a patient in an epileptic fit to the violence of his attendants; while there are others who, unrealised by themselves, have been in the earlier stages of their attack, very violent and dangerous, and who, when they have recovered, have strongly asserted that there was no necessity for their having been sent to the asylum, and either (wittingly or unwittingly) exhibit their resentment by making unfounded complaints."

The report adds that everything possible was done to make the patients happy and relieve tedium. The extensive grounds and gardens, the cricket fields and bowling greens attached to the hospitals were great attractions, and did much to promote the health and vigour of convalescents. Entertainments in the recreation room at each asylum were an inestimable boon.

There were 38,000 nervous cases. "It was desired, as long as possible," states the report, "to avoid certifying these cases of nervous breakdown as lunatics, and it was decided, therefore, to keep them in hospitals under military control as long as the war lasted—afterwards limited to nine months."

The hospitals provided, with some hutments, as many as 27,778 permanent beds, and with extensions could accommodate over 31,000 patients. The number of patients treated in them up to the date of the report, which was May 15 this year, had been 482,949, approximately equivalent to more than one-sixth of the total number of sick and wounded men from all fronts during the war. Of the total of all classes of patients that were treated the number that died, 3,258, was equivalent to only 0.67 per cent.

Commenting upon what it regards as the success of the scheme, the report proceeds: "In the face of these facts it is much to be regretted that severe and unjustified criticisms have been made by persons who have regarded it as derogatory to a mentally disturbed soldier to be placed in a so-called pauper asylum, and who have persistently referred, in disparaging terms, to both the medical and nursing staffs of these institutions."

